# A Probabilistic Re-Intepretation of Confidence Scores in Multi-Exit Models

**DOI:** 10.3390/e24010001

**Published:** 2021-12-21

**Authors:** Jary Pomponi, Simone Scardapane, Aurelio Uncini

**Affiliations:** Department of Information Engineering, Electronics and Telecommunications (DIET), Sapienza University of Rome, 00139 Rome, Italy; simone.scardapane@uniroma1.it (S.S.); aurelio.uncini@uniroma1.it (A.U.)

**Keywords:** branch neural networks, deep learning, deep neural networks, adaptive computation, fast inference

## Abstract

In this paper, we propose a new approach to train a deep neural network with multiple intermediate auxiliary classifiers, branching from it. These ‘multi-exits’ models can be used to reduce the inference time by performing early exit on the intermediate branches, if the confidence of the prediction is higher than a threshold. They rely on the assumption that not all the samples require the same amount of processing to yield a good prediction. In this paper, we propose a way to train jointly all the branches of a multi-exit model without hyper-parameters, by weighting the predictions from each branch with a trained confidence score. Each confidence score is an approximation of the real one produced by the branch, and it is calculated and regularized while training the rest of the model. We evaluate our proposal on a set of image classification benchmarks, using different neural models and early-exit stopping criteria.

## 1. Introduction

Neural networks models are becoming deeper and more complex each year, following the goal of improving the accuracy. For this reason, the training and the inference time are growing, as well. These models, usually designed as sequence of functions, implemented as differentiable layers, are trained by using back-propagation techniques from the last layer inwards. Combining these aspects, some problems can arise when going deeper by adding more layers. In addition, when using deeper architectures, a phenomenon called over-thinking can arise [1,2], where images that are correctly predicted using shallow models can become incorrectly classified with progressively deeper architectures. Many methods proposed to overcome these problems aim to build models that are less linear, e.g., models that use residual connections [3], in a way that the backward information is better propagated, but phenomena, such as overfitting, vanishing gradients, and over-thinking, can still happen [4].

Over-thinking aside, a deeper model usually can extract and learn high-level features better than a shallower one. In fact, going deeper works because it improves the performances by fixing the mistakes made by shallow models [5]. This trend, started with Reference [6], allowed for achieving breakthroughs in many problems, imposing neural networks as state of the art methods in many tasks. However, in recent years, a rich number of contributions, e.g., References [7,8,9], have shown that many of the patterns present in a dataset are repeated, and some samples can be correctly classified also using a shallower architecture. This means that the added capacity is redundant for those samples. This observation has motivated research of models that have some adaptive mechanism, so that the computational graph can be adapted based on the complexity of the input sample. One of these input adaptive mechanisms is called multi-exit strategy [2,10,11], in which the networks are endowed, in addition to the final classifier, of multiple intermediate branches, each one ending with the associated classifier. Multi-exit strategy methods fall into an adaptive category called dynamic depth because the depth reached by each sample is decided using an halting process. Others similar approaches exist, such as dynamic width, where all the layers are used, but the units are selectively activated based on the input sample [12,13,14], or dynamic routing, where a model has multiple inner inference paths, and performs dynamic routing to adapt the computational graph to each sample [15,16,17]. Instead, using a Multi-exit approach, the inference phase is faster and requires less computational power because each auxiliary branch can be used to halt the decision process for a given sample, as opposed to other adaptive models where a sample is passed through all the layers. In this way, samples containing patterns that can be correctly classified in early stages of the network do not require a complete forward process, saving computational resources.

Adding one or two intermediate branches is a known technique for improving gradient propagation in deep networks [6]. Multi-exit networks, however, add many exits and generally train all branch at the same time, using the intermediate branches to perform early exit during the inference phase. Training these models can be difficult due to the multiple losses produced (one for each branch), that must be weighted accordingly to some prior given by the user; this is feasible when working with small networks but becomes more difficult with deeper models. In addition, during the inference phase, separate thresholds must be set for the intermediate exits that depend on their confidence and their accuracy.

In this paper, we propose a training method for multi-exit networks that simplifies the design of these models (in particular, setting all the thresholds relative to the separate auxiliary classifiers) and, simultaneously, improves the computational resources saved during the inference phase.

### Notation

We use calligraphic letters (e.g., F, C) to denote functional blocks, such as sequences of layers in a deep network. Lower-case letters (e.g., *x*, *y*) denote *n*-dimensional arrays, while upper-dimensional letters (e.g., *E*, *L*) denote constants.

## 2. Deep Networks with Early Exits

In this section, we describe the generic neural network model we consider in this work. For a more in-depth overview, we refer to Reference [18]. Denote by (x,y) an input-output pair (e.g., an image and its corresponding label), and, by F(x), a deep network or any other differentiable model. Given a dataset ${\left\{(x_{i},y_{i})\right\}}_{i=0,\cdots N}$ of *N* elements, we train the parameters *w* of the model by minimizing an empirical cost over a given loss function l(·,·) using stochastic gradient descent:

(1) $$L\left(w\right)=\frac{1}{N}\sum_{i}l\left(y_{i},F\left(x_{i}\right)\right)\;.$$

The idea of multi-output networks is to improve training by considering a sequence of intermediate predictions ‘branching’ from mid-points of F. As mentioned in Section 1, this is motivated by the fact that a subset of the model can be sufficient for successfully classifying certain inputs, and intermediate outputs can also provide additional training signals that significantly improve the gradient flow.

To this end, suppose that the original model is decomposed into a sequence of *E* sub-blocks $L_{1},\ldots ,L_{E}$:

(2) $$F=L_{E}\circ L_{E-1}\circ \ldots \circ L_{1}\;,$$

where ∘ denotes function composition. Most deep networks admit a natural decomposition of this form. In particular, note that (2) is assumed to be a sequence of blocks, but the processing inside each block does not have this restriction (e.g., residual connections are allowed as long as a residual connection does not span more than a single block). For readability, we also define ${\bar{L}}_{e}$ as the output of the *e*-th block as:

(3) $${\bar{L}}_{e}=\left\{\begin{array}{cc} L_{1} & \;\mathrm{if}\;e=1\\ L_{e}\circ {\bar{L}}_{e-1} & \;\mathrm{otherwise} \end{array}\right.\;.$$

After each non-terminal sub-block, e=1,…,E−1, we apply a small classifier to obtain an intermediate prediction:

(4) $$F_{e}\left(x\right)=C_{e}\circ \bar{L_{e}}\left(x\right).$$

$C_{e}$ is designed to be as compact as possible to reduce the computational overhead, e.g., a single linear projection, followed by an element-wise nonlinearity. The result is a sequence $F_{1}\left(x\right),\ldots ,F_{E-1}\left(x\right),F\left(x\right)$ of *E* predictions for each input *x*. While it is reasonable to assume that the average accuracy of a branch is higher that the preceding branches, different branches can mistake separate subsets of the original dataset. For this reason, adaptively combining and using the entire set of exits can significantly improve the performance.

A common way to train these models is to optimize over the joint set of losses with respect to all exits:

(5) $$L_{\mathrm{joint}}\left(w\right)=L\left(w\right)+\sum_{e=1}^{E-1}\lambda_{e}\cdot \underset{\mathrm{Loss}\;\mathrm{for}\;\mathrm{the}\;e-\mathrm{th}\;\mathrm{branch}}{\underbrace{\left[\frac{1}{N}\sum_{i}l\left(y_{i},F_{e}\left(x_{i}\right)\right)\right]}}\;,$$

where $\lambda_{e}$ is a hyper-parameter balancing the contribution of the *e*-th loss. A major strength of multi-output networks is that they provide a reasonable way to early-exit the model, and to reduce computational time, by selecting for each input the earliest possible prediction which is assumed to be correct. A common way, popularized by Reference [10], is to compute the entropy of each intermediate prediction. Denoting by ${\hat{y}}_{e}=F_{e}\left(x\right)$, we compute its entropy as:

(6) $$H\left[{\hat{y}}_{e}\right]=-\sum_{c}{\hat{y}}_{e,c}\log\left({\hat{y}}_{e,c}\right)\;,$$

where ${\hat{y}}_{e,c}$ denotes the *c*-th output (the probability for the *c*-th class) of the *e*-th intermediate exit. Then, we consider the prediction correct if its entropy is lower than a user-defined threshold $\gamma_{e}$, $H\left[{\hat{y}}_{e}\right]<\gamma_{e}$.

Note that an input early exiting at the beginning of the network can significantly speedup the computational cost of the inference procedure. However, the procedure described up to this point has a number of shortcomings. Notably, deep networks are notoriously miscalibrated, making the entropy computation potentially misleading (e.g., if a network is over-confident of its own prediction). As a consequence, setting the per-exit thresholds $\gamma_{e}$ is difficult because a user needs to take into consideration both the accuracy and the calibration of each exit [10]. In addition, the inference phase tend to be disjointed from the training phase, potentially creating further mismatches in term of performance.

### Differentiable Branching

Before introducing our method, we briefly describe the training method developed in Reference [11], which we use as starting point. The main idea is that we can let the network itself select the best early exit for a given input, by associating to each auxiliary classifier an additional output value:

(7) $$F_{e}\left(x\right)=\left[\begin{array}{c} {\hat{y}}_{e}\left(x\right)\\ c_{e}\left(x\right) \end{array}\right]\;,$$

where ${\hat{y}}_{e}$ is the auxiliary prediction (as in the previous section), and $c_{e}$ is a *confidence* score in $\left[0,1\right]$ denoting the confidence the network has that exit *e* is correct. We can use these values to soft-combine all auxiliary prediction as:

(8) $${\tilde{y}}_{e}\left(x\right)=c_{e}\left(x\right)\cdot {\hat{y}}_{e}\left(x\right)+\left(1-c_{e}\left(x\right)\right)\cdot {\tilde{y}}_{e+1}\left(x\right),$$

where we define $c_{E}\left(x\right)=1$ to stop the recursion at the end. We can train the network by minimizing (1) over ${\tilde{y}}_{E}$ instead of the original output. During inference, the network early exits whenever the confidence is higher than a pre-defined threshold, generally 0.5, i.e., $c_{e}\geq 0.5$. Although this method can provide significant gains in performance in some scenarios [11], it can still happen that the confidence values are not precise, requiring to fine-tune the exit threshold for each branch. The main idea of this work is to reinterpret the output of the network probabilistically, to provide more precise uncertainty quantification during training.

## 3. Our Proposal

We propose a different optimization schema, which requires no hyper-parameters and, therefore, works without having to manually balance the training of the branches. It also incorporate an exploration strategy of the intermediate exits, that leads to higher accuracy scores, as well as a drastic reduction of inference steps, during the inference phase. This is achieved by using, for each branch, an approximation of the classification’s certainty, using a confidence score also produced by the model, which is regularized during the training.

Starting from (7), we use each confidence score as a parameter for a continuous relaxation of the Bernoulli distribution, called ${\mathrm{BinConcrete}}_{e}\left(\tau ,c_{e}\left(x\right)\right)$ [19,20], with temperature value τ. We use this distribution, instead of the Bernoulli distribution, because the reparameterization trick cannot be applied to a discrete random variable, due to the lacking of differentiable function to transform a base distribution into a discrete distribution. The BinConcrete is a continuous relaxation of the Bernoulli distribution, with support in (0,1), that can be reparameterized, and its sampling procedure can be described, in our case, as:

(9) $$\begin{array}{cc} \; & \left(1)\;U∼Uniform(0,1\right)\\ \; & \left(2\right)\;l=\frac{\log\left(U\right)-\log\left(-U+1\right)+\log\left(c_{e}\right)-\log\left(-c_{e}+1\right)}{\tau }\\ \; & \left(3\right)\;w_{e,x}=\frac{1}{1+\exp(-l)} \end{array}.$$

As the temperature τ converges to 0, the random variable $w_{e,x}$ converges to a Bernoulli with parameter $c_{e}$; as the the temperature goes to *∞*, the distribution of the weight becomes degenerate at 0.5. During the training, we use these distributions to sample the weights associated to the branches, as $w_{e,x}∼{\mathrm{BinConcrete}}_{e}\left(\tau ,c_{e}\left(x\right)\right)$, for e=1.⋯E−1. By using this sampling technique, we are forcing the model to have an exploratory behavior during the training, in such a way that all the branches are used and trained. These weights are also used to create a distribution for each sample *x*, as:

(10) $${\bar{w}}_{e,x}=w_{e,x}\prod_{i=1}^{e-1}\left(1-w_{i,x}\right)\;,$$

which is a valid distribution, having $\sum {\bar{w}}_{e,x}=1$, since the last weight is always 1. This formulation is known as the stick-breaking process [21]. We notice that the resulting distribution does not require the complete forward process to be computed (as opposed, for example, to the softmax function) and can be calculated branch by branch. These weights are used to create the final output of the model, as:

(11) $$y_{f}\left(x\right)=\sum_{e=1}^{E-1}{\bar{w}}_{e,x_{i}}{\hat{y}}_{e}\left(x_{i}\right)\;.$$

**Figure 1:**
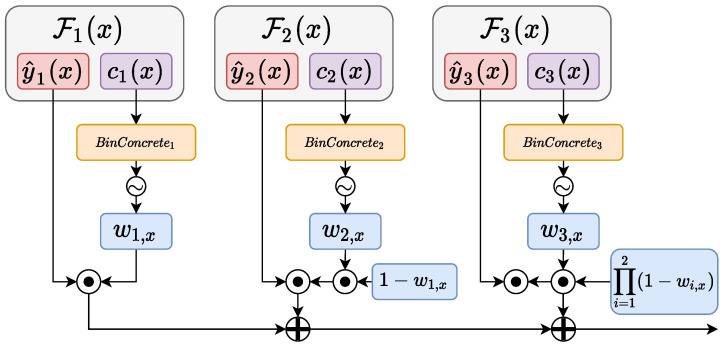
The image shows the overall proposed approach, for the first three branches of a generic model. The modules and the associated operations, with the exceptions of modules ${\hat{y}}_{i}\left(\cdot \right)$ and $c_{i}\left(\cdot \right)$, are all introduced in this paper.

The resulting vector is a combination of all the intermediate branches of a model. In this way, each branch’s prediction is weighted using a normalized version of the confidence score $c_{e}$, produced by the branch itself. The overall procedure to produce $y_{f}\left(x\right)$ is graphically visualized in Figure 1. The final loss is given by:

(12) $$l_{\mathrm{binary}}=\frac{1}{N}\sum_{i}l\left(y_{i},y_{f}\left(x\right)\right)+l\left(y_{i},y_{E}\left(x\right)\right)\;.$$

We divide the loss of the intermediate branches and the one associated to the last layer to avoid that the weight associated to the latter, which can be smaller if the model confidence is high in the early stages of the model, which overshadows its training.

In addition to the exposed procedure, to avoid unexpected behaviors and to force the model to correctly understand when to halt the inference phase, we also want to regularize the confidence scores. To this end, during training, we add a regularization factor to the loss as described in the following section.

### 3.1. Regularization of the Confidence

Given the confidence score of a branch *e* for a given input sample *x*, and a function that returns the most probable class produced by the branch *e*, $p_{e}\left(x\right)=\mathrm{softmax}\;F_{e}\left(x\right)$, the regularization term is calculated as a Binary Cross Entropy between the confidence score and the output of the function 𝟙(·,·):

(13) $$R_{}\left(x,y\right)=\sum_{e=1}^{E-1}-c_{e,x}\log(𝟙\left(y,p_{e}\left(x\right)\right)-\left(1-c_{e,x}\right)\log(𝟙\left(y,p_{e}\left(x\right)\right)\;,$$

where $𝟙(y,p_{e}\left(x\right))$ is a function that returns 1 if the label predicted by the branch *e* is equals to the ground truth label *y* ($p_{e}\left(x\right)=y$); otherwise, it returns 0. Since the function is not differentiable, its outputs are calculated before each training step, without interfering with the gradient computations. The final loss is given by:

(14) $$l_{\mathrm{binary}}=\frac{1}{N}\sum_{i}l\left(y_{i},y_{f}\left(x\right)\right)+l\left(y_{i},y_{E}\left(x\right)\right)+\beta R_{}\left(x_{i},y_{i}\right)\;,$$

where β is a scalar that balances the classification loss and the regularization loss during the training. This is the only hyper-parameter of the proposed approach, but, as we will see in the experimental section, it requires only a small amount of manual tuning.

### 3.2. Inference Phase

During the inference phase, multiple algorithms can be used. Firstly, as exposed before, we can halt the inference at a given branch if the classification entropy is lower than a certain threshold. Secondly, we can exit to a branch that has $c_{e}$ higher than a certain confidence threshold; the latter approach is preferable in our case, assuming that the scores reflect the real confidence of a prediction. Here, both of the approaches can be used, but, to better reflect the proposed training procedure, we introduce a new halting method. Following how the weights are calculated in the Equation (10), we halt the inference phase at the branch *e* if:

(15) $$c_{e,x}\prod_{i=1}^{e-1}\left(1-c_{i,x}\right)\geq \epsilon ,$$

where ϵ is a threshold value bounded in [0,1]. The equation means that we halt the inference process if the cumulative confidence score, calculated using all the confidence scores up to the branch *e*, exceeds a confidence threshold. A similar halting process has been proposed in Reference [22], where a model, called PonderNet, is reused multiple a variable number of times, until the halting criterion is met.

## 4. Experimental Evaluation

### 4.1. Experimental Setup

We evaluate our proposal on three image classification datasets with various number of architectures, from small ones to deeper models. The datasets are: SVHN [23] (it contains 10 classes, and it is composed by 73,257 training samples and 26,032 images used for testing), CIFAR10, and CIFAR100 [24] (these contain, respectively, 10 and 100 classes, and both have 50,000 training images and 10,000 testing images). As architectures, we use AlexNet, VGG11 [25], and ResNet20 [3]. To better understand the impact of our proposal, we train all the combinations dataset-architecture. For each of these combinations, we firstly train the baseline model without intermediate branches. The resulting backbones are used as a starting point for the models with multiple branches. Concerning where to position the branches in the model, we add an intermediate branch after each layer for AlexNet and VGG11, resulting in, respectively, 5 and 8 classifiers (including the last one), while the branches in ResNet20 are placed after each block, resulting in a total of 10 branches. Each branch is composed by a convolutional layer of 128 filters, followed by a max pooling operation, if the dimensions of the image are big enough. This block is followed by a ReLU activation function and a classification linear layer, producing ${\hat{y}}_{e}\left(x\right)$. Regarding our method, in addition to the aforementioned branch architecture, the classification layer also produces a scalar value, that is processed using a sigmoid activation function; this scalar is the confidence score $c_{e}\left(x\right)$.

All the models are trained using the SGD optimizer, with learning rate equals to 0.01 and momentum set to 0.9. The datasets are augmented only during the training of the base model. For each training procedure, we use 10% of the training dataset as development set, and the accuracy score calculated on it is used to save the best model (this is also used as early stopping criteria, when the development score does not increases for 5 consecutive epochs).

We compare our proposal to the joint method [10], that minimizes the sum of the branches losses. For this purpose, following the original paper, we performed a hyper-parameters search to find the best set of weights to balance the losses.

Regarding our proposal, we keep β, in (14), fixed to 1. The temperature is annealed from 20 to 5 exponentially, and then it remains fixed. The temperature scaling, combined with confidence scores sampling, is crucial because it avoids that intermediate branches are ignored during the initial stages of the training process (being their scores close to zero), by forcing the exploration.

The code, including the configuration files used to run the experiments, is available online https://github.com/jaryP/ConfidenceBranchNetwok (accessed on 17 December 2021).

### 4.2. Results and Discussions

We start by analyzing if our proposal can improve the base accuracy obtained by base models without auxiliary classifiers. Table 1 contains the results obtained on all the combination of datasets and architectures. It shows that our approach is capable of improving the results on all the experiments, as opposed to the joint training, which fails to do it with some combination of dataset-architecture. Joint training struggles to achieve better accuracy when the dataset becomes more complex and the architecture deeper. In fact, it is capable of achieving better scores on all the experiments involving SVHN, but starts to fail even on CIFAR10, when the architecture is deep. This is more evident when looking at the results obtained on CIFAR100. This also happens because finding the right hyper-parameters for joint training is hard and computationally expensive, while our method is capable to regularize itself during the training, thanks to the regularization term.

Next, we compare our proposal with joint training when it comes to reducing the inference steps performed. Since the halting process in joint training can be done only using the entropy of the classification, we study how varying a threshold over the prediction’s entropy changes the final results for both the approaches.

To give a complete overview of the gain obtained during the inference phase, that is also independent of the time complexity, we use a novel gain score. To calculate it, firstly, we calculate the normalized cost $C_{e}\in \left[0,1\right]$ ($C_{E}=1$), in terms of operations, required to reach each branch *e* of the model (this cost calculation is based on the one proposed in Reference [11]). Then, given also the percentage of how many samples halted at a given branch *e*, as $h_{e}$, we have the overall gain:

(16) $$G=\sum_{e=1}^{E}\left(1-C_{e}\right)\cdot h_{e}.$$

We use this value, which is bounded G∈[0,1], to visualize how the halting process affects the computational cost required by the inference phase: it is 0 when all the samples reach the final layer, and it is near to zero when all the samples exit at the first layer. Combining this score with various threshold helps us to choice the right one.

**Figure 2:**
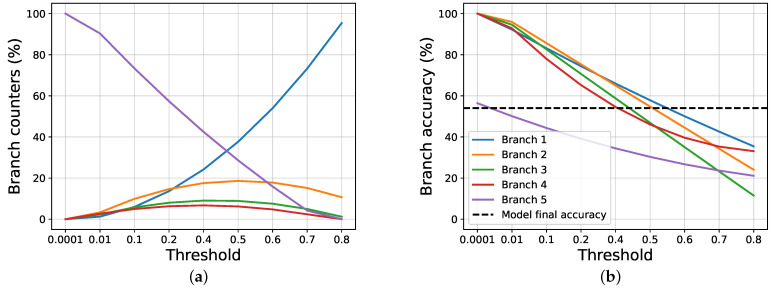
The images show the results obtained by the Joint Training approach on AlexNet trained on CIFAR100, while varying the threshold applied on the entropy. (**a**) Exit counters for each branch of the model. (**b**) Exit accuracy for each branch of the model.

**Figure 3:**
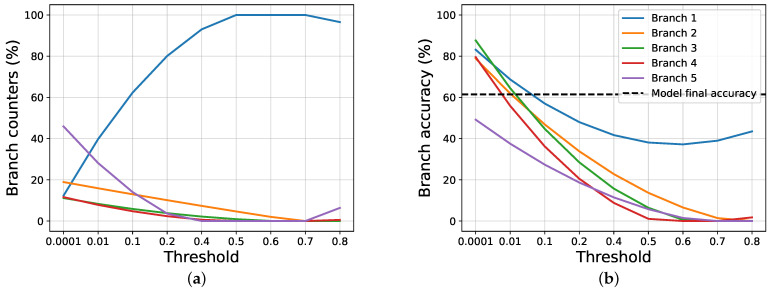
The images show the results obtained by ours proposal on AlexNet trained on CIFAR100, while varying the threshold applied on the entropy. (**a**) Exit counters for each branch of the model. (**b**) Exit accuracy for each branch of the model.

To study the gain, firstly, we compare our proposal against the joint training approach, following the halting techniques exposed in Section 2. To do this, we apply a threshold over the classification entropy of each intermediate classifier, and, if the normalized entropy is lower than, or equal to, the threshold, we halt the inference, and the selected branch is used, without further going deeper; otherwise, we pass to the next branch. If a sample is not halted in any intermediate branch, the last one is used. Then, we study how our halting proposal works. Figure 2 and Figure 3 show, respectively, how the scores and the counters vary for the joint training and our proposal, when a threshold on the entropy is applied on AlexNet trained using CIFAR100.

We notice that, when using a very restrictive threshold, our proposal is able to halt many samples in the intermediate branches, while using joint training almost all samples reach the last layer. This happens because the joint training does not use any information about the certainty of the intermediate classifications, as opposed to out method, that uses a regularization term based on it. When increasing the threshold, the results become similar, both in terms of exit counters and branches scores. The results obtained by the two approaches are very different when it comes to evaluating the overall accuracy and the gain achieved.

**Figure 4:**
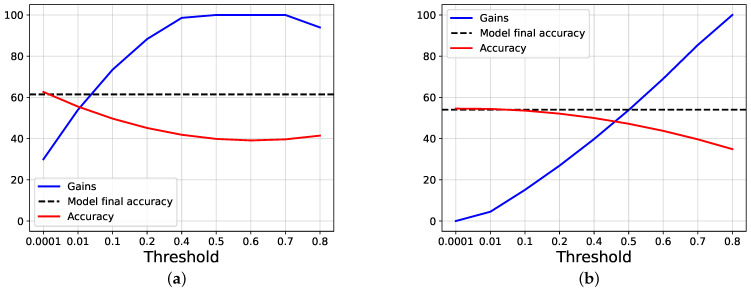
The images show the results, in terms of gain and accuracy, obtained using AlexNet trained on CIFAR100, while varying the threshold applied on the entropy. The dashed lines show the final accuracy reached by the model at the end of the training phase. (**a**) The results obtained by our proposal, while varying the threshold on the entropy. (**b**) The results obtained by Joint trainer, while varying the threshold on the entropy.

Figure 4 shows the overall accuracy obtained by the model while varying the threshold, as well as the gain, calculated as exposed before; as comparison, the accuracy obtained by the models at the end of the training is also shown (which is higher for our proposal). We can see that the accuracy scores start both around the final accuracy obtained by each approach, but the gain is near zero using joint training, while it is 30% using our proposal. When the lines intersect, we have that both our gain and score are around 55%, while, using joint training, both of the values are less than 50%.

**Figure 5:**
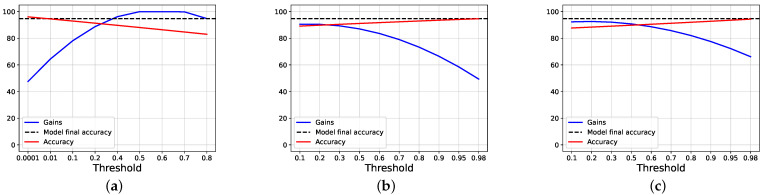
The images show the results, in terms of gain and accuracy, obtained using VGG11 trained on SVHN, while varying the threshold and the halting procedure. The dashed lines show the final accuracy reached by the model at the end of the training phase. (**a**) The results obtained by our proposal, while varying the threshold on the entropy. (**b**) The results obtained by our proposal, while varying the threshold on the confidence scores. (**c**) The results obtained by our proposal, while varying the cumulative threshold.

Next, we study the scores obtained by our proposal when using the proposed halting process. To this end, we select the most interesting result obtained across all the experiments. As before, we evaluate multiple thresholds, to understand how the choice affects the final results, in term of accuracy, branches counter and gain. Firstly, we compare the results obtained using our halting process with the others approaches. To this end, we compare the three halting process in Figure 5. The images show that a result near to the optimum is possible using all the approaches. Looking at the entropy threshold, it happens when using a low threshold on the entropy, while, looking at the other two, it happens using high values. However, in the first case, we reach a gain near 50%, while, using our halting proposal, we have a gain around 70%. This behavior is expected, since, using the proposed halting process, we explicitly use the regularization information also during the inference phase, speeding it.. The binary approach reaches an accuracy score slightly higher than our proposed approach, but the gain drops faster. A downside of our approach, in this case, is that it does not reach a the maximum gain because, during the training, all the branches are explored based on the expected confidence score.

**Figure 6:**
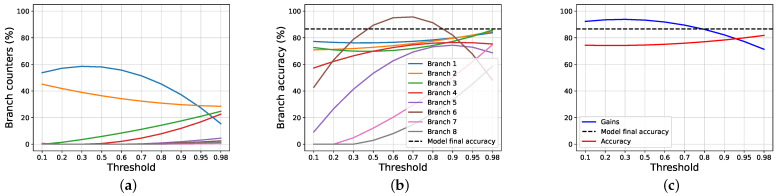
The images show the results obtained by ours proposal on VGG11 trained on CIFAR10, while varying the threshold applied on the cumulative confidence of the model. (**a**) Exit counters for each branch of the model. (**b**) Exit accuracy for each branch of the model. (**c**) Gain and accuracy obtained while varying the cumulative threshold.

**Figure 7:**
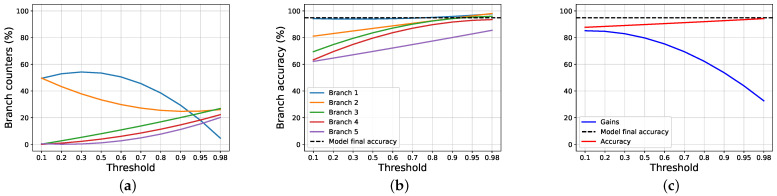
The images show the results obtained by ours proposal on AlexNet trained on SVHN, while varying the threshold applied on the cumulative confidence of the model. (**a**) Exit counters for each branch of the model. (**b**) Exit accuracy for each branch of the model. (**c**) Gain and accuracy obtained, while varying the cumulative threshold.

We can see that the halting process based on the cumulative threshold hurts the accuracy score a little (around 5% of accuracy points are lost), but the gain vantage is remarkable: it goes from 30%, when using the entropy, to 85% using our halting process. More interesting results are shown in Figure 6 and Figure 7. We see that the gain is near the maximum while using low thresholds without hurting the performances substantially, and decreases until it reaches around 75% of gain, while the score is very close to the one obtained by the model. So, if the lost of some accuracy points is acceptable, the gain achieved can be very high.

**Figure 8:**
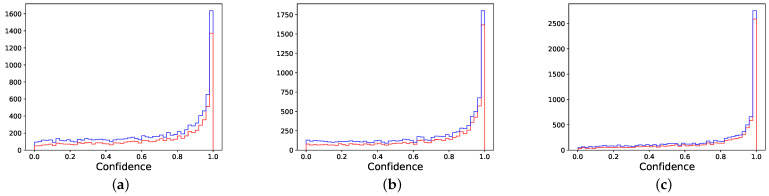
Early-exit cumulative confidence for the first three branches from VGG11 trained on CIFAR10. For each bin, the bottom part is the cumulative confidence of correctly classified samples, and the upper part is associated to the wrongly classified samples. (**a**) Branch 1. (**b**) Branch 2. (**c**) Branch 3.

For completeness, in Figure 8, we report the distribution of cumulative confidence scores for three branches from VGG11 trained on CIFAR10.

### 4.3. Ablation Studies

In this section, we study how the various choices affect the final results achieved by our proposal. Firstly, we start by studying if the regularization term is necessary, and then we check how the results change when we do not sample from the distributions of the confidence scores, and, in the end, we remove both of the techniques. The experiments are based on ResNet20 trained using CIFAR10, and we used our proposed process as halting technique.

**Figure 9:**
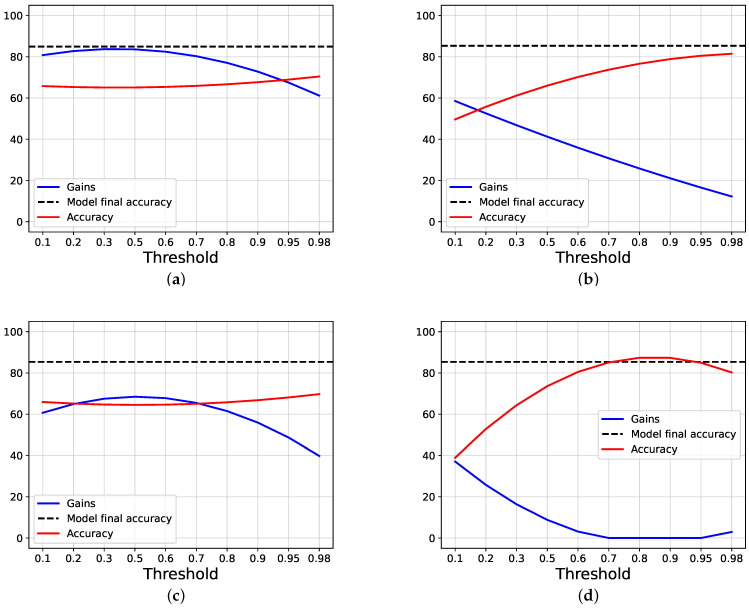
The images show the ablation results, in term of gain and accuracy, while varying the threshold of the halting process proposed. The results are based on ResNet20 trained on CIFAR10. Standard is the training exposed before, while NS and NR mean, respectively, that sampling of the weights is not performed during the training procedure and that the regularization term is neglected while training. (**a**) Standard training. (**b**) NS training. (**c**) NR training. (**d**) NS + NR training.

Figure 9 shows the results obtained. The worst results are achieved when both sampling and regularization methods are neglected. In fact, the accuracy reaches a result near to the optimal one only when the gain is close to zero; similar results are obtained when no sampling is performed. When the regularization term is neglected, the results achieved are better then the ones already exposed, but without reaching the one achieved by the standard training procedure, with the gains being damaged more than the score.

In Table 2, the final accuracy scores for each branch are shown. As expected, the results associated to NR are very close to the one obtained by the standard training procedure. This highlights the fact that the regularization term does not hurt the performances of the model, while it is necessary to achieve a good gain. In addition, as expected, the training without sampling does not explore all the branches because many of them achieve low results.

## 5. Conclusions

In this article, we proposed a novel method to train neural models that have multiple intermediate classifiers, branching off from the main network, with a minimal number of hyper-parameters. The training strategy we proposed is capable of correctly training all the classifiers, reaching higher scores than the plain model with only a classifier in the end. Moreover, our method is capable of speeding up the inference phase without hurting the performances significantly, due to the high number of samples that are correctly classified in the early stages of the models.

In the future, we plan to extend our training method, by designing additional regularization techniques that can help in speeding up the inference phase even more. Moreover, another extension to be considered is to also speed up the training process by halting each sample at an intermediate branch, instead of propagating it through all the neural network, as the current approaches do. We also plan to investigate branching networks on different benchmarks beyond image classification.

## Figures and Tables

**Table 1:** Test accuracy of the three datasets being compared. The best results for each combination of dataset and architecture are highlighted in bold.

Dataset	AlexNet			VGG11			ResNet20		
	Baseline	Joint	Ours	Baseline	Joint	Ours	Baseline	Joint	Ours
SVHN	93.44	94.17	95.49	94.53	95.04	95.40	94.44	95.02	95.13
CIFAR10	88.24	86.27	90.21	86.04	85.67	86.27	83.89	84.61	85.11
CIFAR100	59.57	54.03	61.43	35.01	34.51	38.37	58.62	57.44	58.63

**Table 2:** The table shows the accuracy results for each branch obtained using different training techniques. Standard is the training exposed before, while NS and NR mean, respectively, that sampling of the weights is not performed during the training procedure and that the regularization term is neglected while training.

Method	Branch Number									
	1	2	3	4	5	6	7	8	9	10
Standard	63.67	66.25	68.30	69.72	74.98	77.3	75.56	79.92	82.92	85.11
NS	10.26	14.79	10.32	32.30	41.58	38.26	36.90	16.61	24.74	84.36
NR	63.22	65.19	64.33	66.81	69.43	69.92	72.78	76.71	77.8	85.06
NR + NS	12.23	10.00	10.00	14.47	18.30	32.04	12.06	10.36	83.99	84.36

## Data Availability

Data sharing not applicable.

## References

[1] Wang W., Shen J. (2017). Deep visual attention prediction. IEEE Trans. Image Process..

[2] Kaya Y., Hong S., Dumitras T. Shallow-deep networks: Understanding and mitigating network overthinking. Proceedings of the 2019 International Conference on Machine Learning (ICML).

[3] He K., Zhang X., Ren S., Sun J. Deep residual learning for image recognition. Proceedings of the IEEE Conference on Computer Vision and Pattern Recognition.

[4] Jastrzębski S., Kenton Z., Arpit D., Ballas N., Fischer A., Bengio Y., Storkey A. (2017). Three factors influencing minima in sgd. arXiv.

[5] Huang G., Chen D., Li T., Wu F., van der Maaten L., Weinberger K.Q. (2017). Multi-scale dense networks for resource efficient image classification. arXiv.

[6] Szegedy C., Liu W., Jia Y., Sermanet P., Reed S., Anguelov D., Erhan D., Vanhoucke V., Rabinovich A. Going deeper with convolutions. Proceedings of the IEEE Conference on Computer Vision and Pattern Recognition (CVPR).

[7] Wang G., Xie X., Lai J., Zhuo J. (2017). Deep Growing Learning. Proceedings of the 2017 IEEE International Conference on Computer Vision (ICCV).

[8] Marquez E.S., Hare J.S., Niranjan M. (2018). Deep cascade learning. IEEE Trans. Neural Netw. Learn. Syst..

[9] Belilovsky E., Eickenberg M., Oyallon E. (2019). Greedy layerwise learning can scale to imagenet. International Conference on Machine Learning.

[10] Teerapittayanon S., McDanel B., Kung H.T. (2016). Branchynet: Fast inference via early exiting from deep neural networks. Proceedings of the 2016 23rd International Conference on Pattern Recognition (ICPR).

[11] Scardapane S., Comminiello D., Scarpiniti M., Baccarelli E., Uncini A. (2020). Differentiable branching in deep networks for fast inference. Proceedings of the 2020 IEEE International Conference on Acoustics, Speech and Signal Processing (ICASSP).

[12] Hua W., Zhou Y., De Sa C., Zhang Z., Suh G.E. Channel gating neural networks. Proceedings of the 33rd International Conference on Neural Information Processing Systems.

[13] Lin J., Rao Y., Lu J., Zhou J., Guyon I., Luxburg U.V., Bengio S., Wallach H., Fergus R., Vishwanathan S., Garnett R. (2017). Runtime Neural Pruning. Advances in Neural Information Processing Systems.

[14] Davis A., Arel I. (2013). Low-rank approximations for conditional feedforward computation in deep neural networks. arXiv.

[15] Odena A., Lawson D., Olah C. (2017). Changing model behavior at test-time using reinforcement learning. arXiv.

[16] Frosst N., Hinton G. (2017). Distilling a neural network into a soft decision tree. arXiv.

[17] Ioannou Y., Robertson D., Zikic D., Kontschieder P., Shotton J., Brown M., Criminisi A. (2016). Decision forests, convolutional networks and the models in-between. arXiv.

[18] Scardapane S., Scarpiniti M., Baccarelli E., Uncini A. (2020). Why should we add early exits to neural networks?. Cogn. Comput..

[19] Maddison C., Mnih A., Teh Y. The concrete distribution: A continuous relaxation of discrete random variables. Proceedings of the International Conference on Learning Representations. International Conference on Learning Representations.

[20] Jang E., Gu S., Poole B. (2016). Categorical reparameterization with gumbel-softmax. arXiv.

[21] Paisley J.W. (2010). A Simple Proof of the Stick-Breaking Construction of the Dirichlet Process.

[22] Banino A., Balaguer J., Blundell C. (2021). Pondernet: Learning to ponder. arXiv.

[23] Netzer Y., Wang T., Coates A., Bissacco A., Wu B., Ng A.Y. (2011). Reading Digits in Natural Images With Unsupervised Feature Learning. http://ufldl.stanford.edu/housenumbers/.

[24] Krizhevsky A., Hinton G. (2009). Learning Multiple Layers of Features from Tiny Images. https://www.cs.toronto.edu/~kriz/cifar.html.

[25] He Y., Zhang X., Sun J. Channel Pruning for Accelerating Very Deep Neural Networks. Proceedings of the IEEE International Conference on Computer Vision.

